# Differential activation of the frontal pole to high vs low calorie foods: The neural basis of food preference in Anorexia Nervosa?

**DOI:** 10.1016/j.pscychresns.2016.10.004

**Published:** 2016-12-30

**Authors:** Jessica C. Scaife, Lauren R. Godier, Andrea Reinecke, Catherine J. Harmer, Rebecca J. Park

**Affiliations:** Department of Psychiatry, University of Oxford, Warneford Hospital, Oxford OX3 7JX, UK

**Keywords:** Reward, Neuroimaging, Psychophysiological interactions, Somatosensory, Cognitive control, Eating disorders

## Abstract

Neuroimaging studies in anorexia nervosa (AN) suggest that altered food reward processing may result from dysfunction in both limbic reward and cortical control centers of the brain. This fMRI study aimed to index the neural correlates of food reward in a subsample of individuals with restrictive AN: twelve currently ill, fourteen recovered individuals and sixteen healthy controls. Participants were shown pictures of high and low-calorie foods and asked to evaluate how much they wanted to eat each one following a four hour fast. Whole-brain task-activated analysis was followed by psychophysiological interaction analysis (PPI) of the amygdala and caudate. In the AN group, we observed a differential pattern of activation in the lateral frontal pole: increasing following presentation of high-calorie stimuli and decreasing in during presentation of low-calorie food pictures, the opposite of which was seen in the healthy control (HC) group. In addition, decreased activation to food pictures was observed in somatosensory regions in the AN group. PPI analyses suggested hypo-connectivity in reward pathways, and between the caudate and both somatosensory and visual processing regions in the AN group. No significant between-group differences were observed between the recovered group and the currently ill and healthy controls in the PPI analysis. Taken together, these findings further our understanding of the neural processes which may underpin the avoidance of high-calorie foods in those with AN and might exacerbate the development of compulsive weight-loss behavior, despite emaciation.

## Highlights

• We compared neural responses to high vs low calorie food pictures in restrictive AN.
• We used both fMRI whole brain and PPI analyses.
• Frontal pole hyper-activated by high calorie foods in AN, the opposite to controls.
• Reduced functional connectivity in reward patthways and somatosensory regions in AN.

## 1 Introduction

Anorexia nervosa (AN) is a severely debilitating psychiatric disorder of unknown aetiology, characterised by the relentless pursuit of self-starvation, leading to severe emaciation (American Psychiatric Association, 2013). There is clear evidence of biological influences and significant heritability (Boraska et al., 2014, Bulik et al., 2006). It has a stereotypical presentation, predominantly in females and a narrow age range of onset (American Psychiatric Association, 2013). AN has low rates of full recovery and a paucity of evidence-based treatments (McKnight and Park, 2010, Watson and Bulik, 2012 National Institute for Health and Care Excellence, 2004). In the quest to develop novel interventions, there is increasing interest in the neurobiological factors underlying AN (Kaye et al., 2009, Kaye et al., 2013b).

Individuals with AN are able to maintain extreme dietary restriction despite malnutrition, and experience intense reward from the pursuit of thinness (Park et al., 2014), which becomes compulsive (Godier and Park, 2014, Godier and Park, 2015a, 2015a). Despite this, AN is characterised by a preoccupation with food stimuli (Cowdrey et al., 2013b, Park et al., 2011, Park et al., 2012). Indeed, an attentional bias for food stimuli is reported in AN, using behavioural tasks such as the Emotional Stroop Task, Visual Dot Probe tasks, and Startle Reflex paradigms (Aspen et al., 2013, Brooks et al., 2011b).

Neuroimaging studies in AN indicate differences in the neural processing of food stimuli in regions associated with reward, somaotosensory input, and cognitive control compared to healthy controls. A popular neural model of AN is one describing decreased activation in ‘bottom up’ limbic regions associated with appetite and reward, in conjunction with increased activation in ‘top down’ prefrontal regions associated with cognitive control (Kaye et al., 2009, van Kuyck et al., 2009). It is suggested that this imbalance underpins a pathological need for thinness and avoidance of food (Kaye et al., 2009). Whilst studies have consistently reported increased activation in cognitive control regions, such as the dorsal prefrontal cortex (DLPFC), and medial prefrontal cortex (MPFC) (Brooks et al., 2011a, Cowdrey et al., 2011), inconsistent results (outlined below) in limbic regions such as the insula and striatum (ventral striatum, caudate/putamen), and amygdala put this suggestion into question. Furthermore, AN patients show reduced delay discounting compared to healthy controls, indicative of excessive self-control, not limited to food stimuli (Steinglass et al., 2012).

The insula processes taste and the incentive value of food, and has dense connections to the striatum (Nunn et al., 2011), a region associated with the generation of motivated/reward-related behavior (Kelley, 2004, Robbins and Everitt, 1996). The amygdala is part of a telencephalic circuit, which also includes the hippocampus, prefrontal cortex and the lateral hypothalamus, and integrates feeding, reward, and motivation (Petrovich et al., 2002). Functional alterations in these regions in AN have therefore been linked to altered food reward processing in this group (Nunn et al., 2011). Some fMRI studies have reported reduced activation of these regions in AN (Holsen et al., 2012, Oberndorfer et al., 2013, Wagner et al., 2008), while others have found increased activation in response to food in acute and recovered AN (Cowdrey et al., 2011, Ellison et al., 1998, Kim et al., 2012). Increased caudate activation is also reported in response to food pictures in acute and recovered AN (Sanders et al., 2015). This has been associated with compulsive traits in underweight AN and may contribute to the persistence of dietary restriction in this group (Rothemund et al., 2011). Furthermore, in a fMRI study incorporating both the taste and sight of food we found that individuals recovered from AN demonstrated heightened insula, striatal (both ventral striatum, and caudate/putamen) and prefrontal activation to both pleasurable and aversive food stimuli (Cowdrey et al., 2011). We suggest that the enhanced response in limbic regions in AN reflects the increased salience of food, which may be appraised as threatening. We suggest that energy-dense food cues increase early activation of prefrontal regions subserving higher order decision-making and cognitive control, which may serve to limit food intake and maintain dietary restriction in AN, despite extreme starvation (Cowdrey et al., 2013a, Cowdrey et al., 2011 Park et al., 2014). This maladaptive response to high-calorie food in the face of starvation may result from dysfunction in regions subserving interoceptive and somatosensory processing of food stimuli in AN, such as the insula and amygdala (Holsen et al., 2012, Joos et al., 2011). These regions may not effectively process information regarding biological needs, despite malnourishment.

Enhanced motivational salience for energy-dense food, such as chocolate, perceived in healthy controls as rewarding (Cowdrey et al., 2011) may of course be in conflict with a conscious desire to be thin, such that subcortical desires can become dreads (Berridge et al., 2009, Zhang et al., 2009). Consistent with this notion, behavioural and neuroimaging evidence suggests that individuals with AN process high and low-calorie food stimuli differentially, and differently from controls - which may in part explain the under-consumption of high-calorie and over-consumption of low-calorie food that characterizes AN. For example, in a previous study by our group (Cowdrey et al., 2013a) implicit and explicit food preferences were compared between four groups of individuals: underweight AN, weight-restored AN, fully recovered AN and controls. Both underweight and weight restored AN groups implicitly and explicitly wanted high-calorie foods less, and low-calorie foods more, an inverted pattern to that seen in healthy controls. Those with restrictive AN also showed less explicit liking of high-calorie food compared to controls. These results contribute to the emergent evidence that abberant food reward processing may contribute to the under-consumption of energy-dense food seen in AN (Cowdrey et al., 2013a, Cowdrey et al., 2011, Park et al., 2014, Wierenga et al., 2015). They are also consistent with differences in the neural response to high vs. low-calorie foods in reward processing regions in AN (Ellison et al., 1998, Rothemund et al., 2011). They also point to the importance of differentiating between high and low-calorie foods in understanding the mechanisms underpinning self-starvation behavior in AN.

The main aim of the current study was to explore the specific prediction, based on the above findings, that in AN as compared to controls there is increased differential activation of the prefrontal cortex to high-calorie compared to low-calorie food pictures. Based on our behavioural findings (Cowdrey et al., 2013a), we predicted increased activity in the prefrontal cortex during high compared to low-calorie food pictures, reflecting increased cognitive control in this condition. Furthermore, given prior research (Cowdrey et al., 2011, Kaye et al., 2013a), we predicted that limbic regions: caudate, insula, and amygdala, often shown to be dysfunctional in AN, would show hypoconnectivity with prefrontal cortical regions, reflecting an imbalance of limbic and frontal control circuits responsible for reward and cognitive control processes in AN (Kaye et al., 2009, Kaye et al., 2013a). We further predicted deficits in somatosensory regions in response to food cues and reduced connectivity between these regions and areas involved in food reward (Favaro et al., 2012). We predicted that these findings would be less extreme in those recovered from AN than in those currently ill.

To test these predictions we used an fMRI adaptation of the food pictures paradigm developed previously, the Leeds Oxford Food Preference Questionnaire (LOFPQ) (Cowdrey et al., 2013a) to index the neural correlates of food reward in AN. Participants completed the LOFPQ and viewed high and low-calorie food pictures under fMRI, whilst focusing on their wanting for each of the food types. As starvation *per se* is associated with severe alterations in cognitive and physiological systems (Cowdrey et al., 2011, Kaye et al., 2009, Wagner et al., 2008), we opted to recruit both individuals currently ill and those fully recovered from AN, and to compare their performance to healthy controls. Further, due to differences reported in response to food stimuli between the subtypes of AN (Brooks et al., 2012), and our particular focus on the neural mechanisms of restrictive behavior in AN, we recruited only those with the restrictive subtype.

We used a psychophysiological interaction analysis (PPI) to investigate the task-specific changes in the relationship between different brain regions in response to food stimuli. In PPI, regions are identified which show increased or decreased activation in relation to seed regions of interest (ROI) (O'Reilly et al., 2012) (Kaye et al., 2009, Kaye et al., 2013a).

## 2 Methods

### 2.1 Participants

Forty-two female participants were recruited into three experimental groups: 12 individuals with a current DSM-IV (American Psychiatric Association, 2000) diagnosis of restrictive anorexia nervosa (AN group), 14 individuals recovered from restrictive AN (AN-R group), and 16 healthy controls (HC group) matched for age and IQ (Table 1). All participants were aged between 18 and 60 years and were right handed. Inclusion criteria for the HC group were a normal Body Mass Index (BMI; 18.5–25), scores within one standard deviation of the global mean scores for young women from the Eating Disorder Examination Questionnaire (EDE-Q)(Fairburn and Beglin, 2008), no evidence of current or past psychiatric disorder, no first-degree relative with a current or past eating disorder diagnosis and no history of neurological or other significant medical illness. Inclusion criteria for the AN-R group included a previous DSM-IV diagnosis of restrictive AN, a BMI within normal range (18.5–25), no significant eating disorder pathology for the 12 months prior to the study and EDE-Q scores within one standard deviation of global mean scores for young women. Inclusion criterion for the AN group included a current DSM-IV diagnosis of restrictive AN, required to be their primary diagnosis.

In the AN group, although AN was the primary diagnosis, two participants also fulfilled criteria for comorbid depression and four were both comorbidly depressed and suffering from Generalised Anxiety Disorder (GAD). One participant was currently being prescribed SSRI/SNRI antidepressant drugs, one was taking atypical antipsychotic drugs and four were taking both.

None of the participants in the AN-R group currently presented with comorbid psychiatric diagnoses. Whilst suffering from AN, twelve were comorbidly depressed, and four were suffering from GAD. Three participants in the AN-R group were taking SSRI/SNRI antidepressant drugs.

Ethical permission for this study was obtained from the NRES South Central – Oxford A Research Ethics Committee (13/SC/0395).

### 2.2 Procedure

Participants were initially asked to complete a number of screening questions, and an online version of the EDE-Q to ensure they met the criteria for the study. Participants were then invited to an initial screening session at the Warneford Hospital. After informed consent was taken, participants were screened for current DSM-IV Axis-I and given a battery of questionnaires to complete. These could also be completed online prior to the session. Height and weight were taken to calculate BMI.

Participants attended a second session at the Oxford Centre for Magnetic Resonance Imaging (OCMR) to complete the fMRI scan and the behavioural task. On the day of testing, participants were asked not to eat for 4 h prior to the fMRI scan and to drink only water or calorie-free drinks (with the exception of two patients who were currently ill and on a meal plan, who consumed a 100 cal cereal bar two hours prior to the scan). Participants completed Visual Analogue Scales (VAS) for the dimensions ‘I am hungry’, ‘how do you see yourself’ (very thin-very fat) and ‘fear of weight gain’ (0–100 mm, not at all – extremely) to assess feelings about body image.

### 2.3 Questionnaire measures

The Structured Clinical Interview for the DSM-IV (American Psychiatric Association, 2000) was used to screen for Axis-I disorders. Eating disorder psychopathology was measured using the Eating Disorder Examination (EDE) (Fairburn et al., 2008) and EDE-Q. Participants completed the Yale-Brown-Cornell Eating Disorder Scale Self-Report Questionnaire (YBC-EDS-SRQ) (Mazure et al., 1994), *which indexes* eating-related preoccupations and/or rituals. Depressive symptoms were measured by using the Beck Depression Inventory (BDI-II) (Beck, 1996). Anxiety symptoms were measured by using the State-Trait Anxiety Inventory (STAI) (Spielberger, 1983). Verbal IQ was measured using the National Adult Reading Test (NART) (Nelson, 1991), with non-native English speakers excluded from group means.

### 2.4 Behavioural task – leeds oxford food preference questionnaire

Components of food reward were assessed using a behavioural task, the Leeds-Oxford food preference questionnaire (LOFPQ; for details see our previous publication (Cowdrey et al., 2013a). Separate measures of liking (hedonic pleasure) and explicit wanting (incentive salience) were assessed by using food stimuli varying along the dimensions of calorie content (high or low) and taste (savoury or sweet). Explicit wanting and liking were assessed using 100 mm VAS scales, responding to the questions “How much do you want some of this food now? ” and “How pleasant would it be to experience the taste of this food now? ”, respectively. Implicit wanting was indexed using reaction times to a behavioural forced-choice component of the task.

### 2.5 fMRI food pictures task

**Fig. 1:**
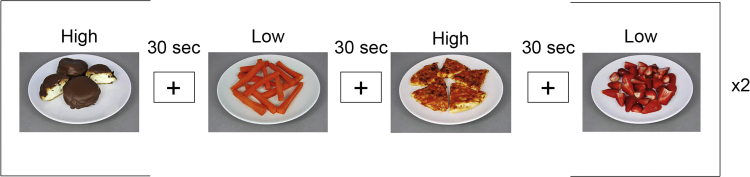
Eight 30 s blocks, 4 high, 4 low-calorie, 30 s fixation cross between blocks. Participants were instructed to ***‘**Focus on how much you want each of the different foods right now’*. Figure shows four example blocks.

Forty high and forty low-calorie food pictures (detailed in Supplementary Information) were presented in 8 blocks of 5 images, with a variable inter-trial interval of between 0.5 and 1.5 s (mean 1 s) in which a crosshair appeared on the screen. Picture blocks alternated with fixation baseline blocks of 30 s See Figure. 1. Prior to the task, participants were instructed to ‘focus on how much you want each of the different foods, right now’. In order to encourage the individuals to focus their attention on the centre of the pictures, a small dot appeared in the centre of the screen, superimposed on the food pictures, 2 – 3.5 s following onset of the picture. Participants were instructed to respond with a button press when they saw the dot in order to focus their attention on the food image. A significant number of button press data were lost, and this was not analysed. This is a limitation of the study, as this data would have provided corroborating evidence that participants were indeed looking at the image.

### 2.6 Neuroimaging protocol

#### 2.6.1 Image acquisition and preprocessing

Scanning was performed at the University of Oxford, Centre for Clinical Magnetic Resonance Research (OCMR) using a 3 T Siemens Trio scanner with a 32 channel head-coil. The neuroimaging protocol comprised functional and structural sequences as follows.

Functional imaging data were analysed using FEAT 6.0, part of FSL (FMRIB Software Library; www.fmrib.ox.ac.ul/fsl) with Z=2.0 and *p*<0.05, including multiple-comparison corrections. T_2_*-weighted functional data were acquired for a whole-brain field-of-view (64×64×40 matrix, voxel resolution 3.0 mm^3^, repetition time (TR)=3000 ms, echo time (TE)=30 ms, flip angle=90°). Field maps were acquired using a dual echo 2D gradient echo sequence with echos at 5.19 and 7.65 ms, and a repetition time of 500 ms. High-resolution T_1_-weighted images were acquired for subject alignment, using an MPRAGE sequence (174×192×192 matrix, voxel resolution 1 mm^3^, TR=2040 ms, TE=4.7 ms, inversion time (TI)=900 ms). T_2_ pre-processing included motion correction (Jenkinson et al., 2002), non-brain removal (Smith, 2002), spatial smoothing (Gaussian kernel FWHM=5.0 mm), grand-mean intensity normalisation of the entire 4D dataset by a single multiplicative factor. Registration to high resolution structural and/or standard space images was carried out using FLIRT (Jenkinson et al., 2002; Jenkinson and Smith, 2001). Registration from high resolution structural to standard space was then further refined using FNIRT nonlinear registration (Andersson et al., 2007a, Andersson, 2007b). A maximum threshold of 3 mm relative motion was applied and absolute and relative motion were extracted for each participant. One-way ANOVA was performed on absolute and relative motion values to determine any group differences.

### 2.7 Task-activated analysis

At the first level, data were analysed using a general linear model approach with local autocorrelation correction (Woolrich et al., 2001). Two regressors of interest (high-calorie, low-calorie) were included. Global GM volume was added as a confound regressor (nuisance) to the GLM design matrix. Fixation blocks were used as the implicit baseline reference.

Contrast images were calculated for picture blocks in general, high-calorie blocks, low-calorie blocks, high versus low-calorie, and low versus high. These individual activation maps were then entered into the group level (HC, AN-R, AN), using a mixed-effects analysis across the whole brain (Beckmann et al., 2003). In the design matrix, contrasts of interest were HC vs AN, HC vs AN-R and AN vs AN-R at the higher level and responses to high vs low-calorie food pictures and all food pictures (high and low meaned) at the lower level.

Significant whole-brain interactions were explored by i) extracting percentage signal changes within these areas and entering these into Group×Task mixed-design ANOVAs and appropriate follow-up *t*-tests, and ii) running Pearson's correlation analyses for the percentage signal change and specific clinical or behavioural measures of relevance to the function of those areas in question and measures of psychopathology (global EDE, YBCEDS past and present and food reward (implicit and explicit wanting and liking in the Leeds Oxford task). Statistical analyses of non-imaging variables were carried out using SPSS software (SPSS, Inc., Chicago IL) version 22.0. Threshold for statistical significance was set to *p*<0.05. Post-hoc results were adjusted for multiple comparisons using Bonferoni corrections. Relative motion did not exceed 3 mm for any participant. There were no significant between-group differences in absolute or relative motion p>0.5.

### 2.8 Connectivity analyses

The left and right amygdala, insula and caudate were chosen as seed regions for connectivity analyses. Seed ROI were identified by initially running small volume correction in these anatomical areas (created from the MNI structural atlas in FSL) and extracting significant clusters from the pictures versus baseline contrast across groups. Significant functional clusters were only identified in the anatomical left amygdala (peak: −22,−4,−12; *Z*=6.7), bilateral insula (left −42, 14, 2; *Z*=6.27, right 32, 26, 6; *Z*=5.89) and bilateral caudate (left −18, 4, 14; *Z*=6.35, right 18, 4, 14; *Z*=6.29) masks. No significant activation was seen in the right amygdala. For each of these five significant clusters and each participant, we extracted a deconvolved time series. These time courses were entered in five separate FSL psychophysical interaction (PPI) analyses, separately for each ROI, along with the two psychological regressors (high-calorie, low-calorie) and the two PPI regressors (high-calorie×time-series, low-calorie×time-series). Global GM volume was added as a confound regressor (nuisance) to the GLM design matrix.

These individual contrast images were then entered into the group level, using a mixed-effects analysis across the whole brain, in order to identify brain areas that showed activation that covaried more strongly with that of the left amygdala or the left or right caudate in one of the three groups during high-calorie blocks, low-calorie blocks, or food-picture blocks in general. Significant interactions were explored by i) extracting BOLD signal changes within these areas and entering these into independent samples *t*-tests, and ii) running Pearson's correlation analyses for the BOLD signal change and the global EDE, YBC-EDS-SRQ (past and present score) and food reward (implicit and explicit wanting and liking in the LOFPQ task). Results were adjusted for multiple comparisons using Bonferoni corrections. Extracted BOLD signal values in the PPI analysis were correlated with mean relative motion and absolute motion.

### 2.9 Structural MRI analysis

For details of structural MRI analysis, see Supplementary Information. Whole brain analysis was carried out using a voxel-based morphometry-style analysis (FSL-VBM) (Douaud et al., 2007) with default settings as described at www.fmrib.ox.ac.uk/fsl/fslvbm/.

## 3 Results

### 3.1 Demographic and psychological characteristics

Table 1 shows the demographic and psychological characteristics of the three experimental groups. The minimum and maximum ages and BMI for the three groups are as follows: HC 19–40 yrs, BMI: 18.8–25, AN-R 19–45 yrs BMI: 18.6–24.7, AN 22–39 yrs, BMI: 12.8–18.8.

### 3.2 LOFPQ results

Full results in the Supplementary Information, Table 4. Mean ratings of implicit wanting, explicit wanting and explicit liking did not significantly differ between the sweet and savoury food categories (*p*>0.05), and thus these categories were collapsed. Data were analysed using repeated measures ANOVA (Calorie × Group) with STAI and BDI scores added as covariates, followed by one-way ANOVAs with Bonferroni corrections.

A significant Calorie × Group interaction was seen for all measures (implicit wanting, *F*=4.4, df=2/39, *p=*0.019; explicit wanting, *F*=4.4, df=2/39, *p=*0.02; liking, *F*=4.1, df=2/39, *p=*0.025). Significant group differences were seen in the high-calorie condition (implicit wanting, *F=*14.21, *df* =2/39, *p*<0.0001; explicit wanting, *F=*11.46, *df* =2/39, *p*<0.0001; liking, *F=*9.36, *df*=2/39, *p*<0.0001), reflecting a reduction in these scores in the AN and AN-R groups compared to the HC group (p<0.05). There were no group differences in implicit or explicit wanting in the low-calorie condition (p>0.05). However, there was a significant group effect in explicit liking for low-calorie foods, (*F*=9.36, df=2/39, p<0.0001), which was driven by increase in liking in both the AN-R group (*p=*0.017) and the AN group (*p=*0.001).

### 3.3 Structural MRI results

In the AN group, there was a widespread reduction in grey matter (GM) with a total reduction of 7%, compared with HC group. Percentage cerebrospinal fluid (CSF) volume was also increased by 13% in the AN group, compared with HC. There were no significant differences in global GM or % CSF between the AN-R and HC groups. White matter showed no group differences. Full results in the Supplementary Information, Table 5.

### 3.4 Functional MRI results

#### 3.4.1 Main effect of task (food pictures vs baseline)

**Fig. 2:**
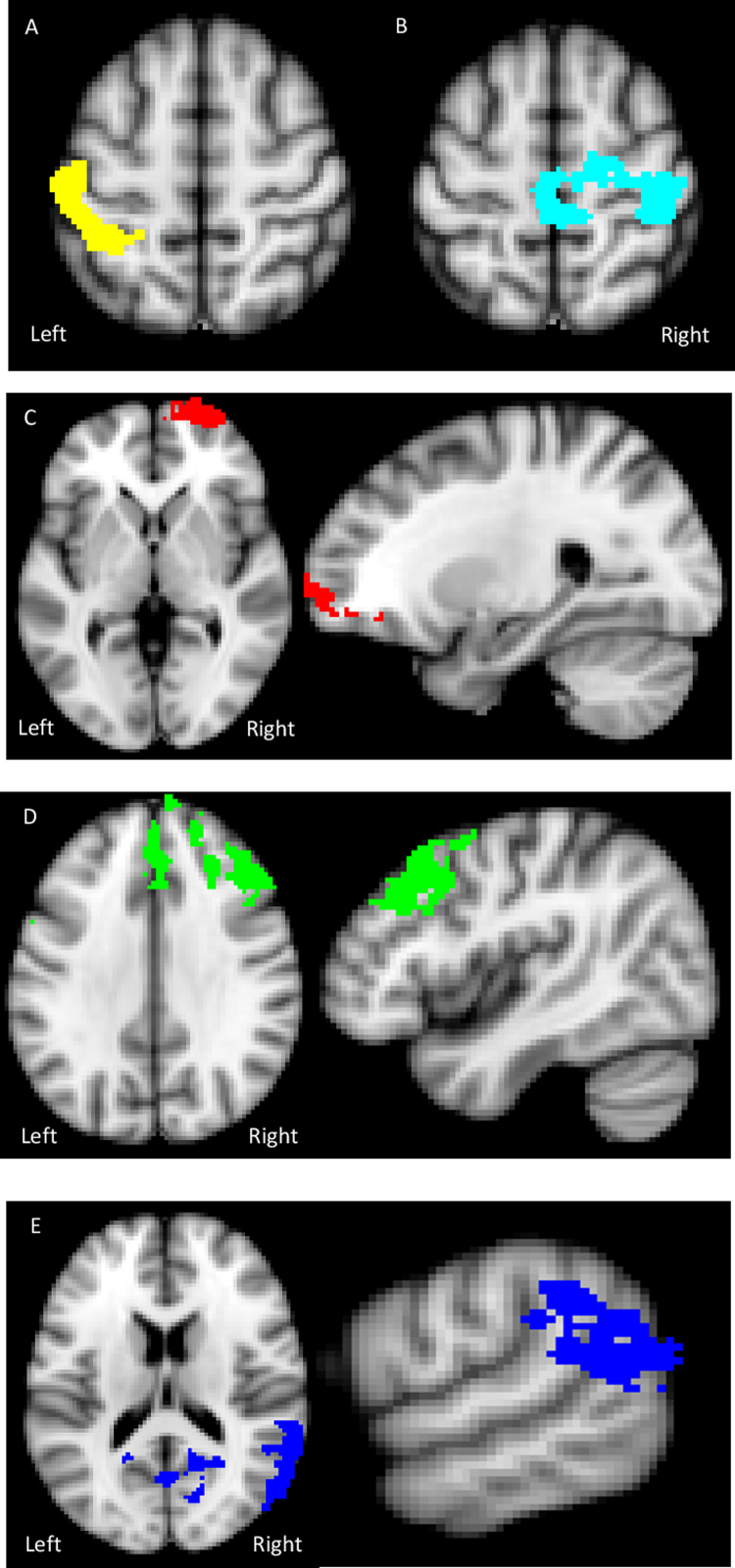
Axial images showing significantly reduced activation in a) the left superior parietal lobule-postcentral gyrus and b) the right postcentral gyrus – precuneus in the food pictures vs baseline condition in the AN group compared to the HC. Axial and coronal images of the frontal pole showing hyper-activation to the high-calorie and hypo-activation to low calorie food pictures in the AN group compared to the HC. Axial and sagittal images showing significantly reduced activation in d) the prefrontal cortex (DMPFC and right DLFC) and e) the angular gyrus-supramarginal gyrus to low calorie food pictures in the AN group compared to the HC.

**Fig. 3:**
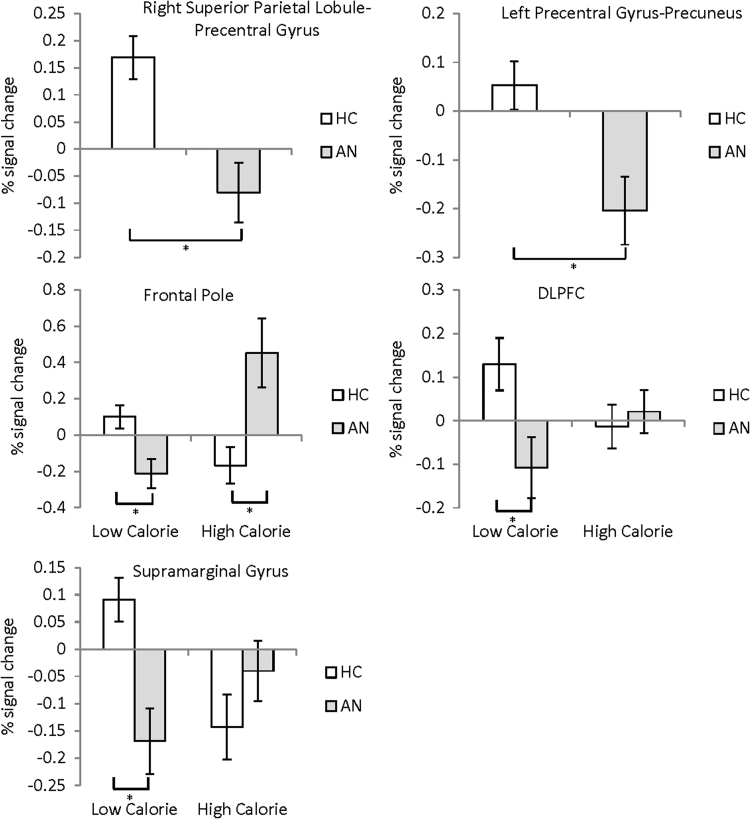
Top row: In the food pictures vs baseline condition, reduced activation was observed in a) the right superior parietal lobule-precentral gyrus and b) the left precentral gyrus-precuneus in the AN group compared to HC. Bottom two rows: High vs Low-calorie food pictures. c) Frontal pole: In the high-calorie condition there was an increased response in the AN group compared to HC. In the low-calorie condition there was a decreased response in the AN group compared to HC. d) In the low-calorie condition there is a decreased response in the DLPFC 10 mm sphere ROI in the AN group compared to HC. e) In the low-calorie condition there is decreased response in the supramarginal/lingual gyri in the AN group compared to HC.

There was a reduction in activation in the right postcentral gyrus-precuneus, extending into the posterior cingulate and in the left superior parietal lobule-postcentral gyrus in the AN group compared to controls, see Table 2 and Fig. 2, Fig. 3. There was no significant reduction in activation in the AN-R group compared to healthy controls.

#### 3.4.2 Calorie × group interaction (High versus Low)

Results showed a significant Calorie × Group interaction in three regions fully detailed in Table 2, Fig. 2, Fig. 3. Firstly, in the right lateral frontal pole, the AN group showed both increased activation to high-calorie pictures compared to HC, and reduced activation to low-calorie pictures compared to HC. Within the AN group, functional activation in the frontal pole in the low-calorie condition significantly negatively correlated with current YBC-EDS-SRQ score (*r*=−0.751, *p*<0.005). Secondly, a large functional cluster was found in the prefrontal cortex, so a 10 mm ROI sphere was used to extract the percentage signal change from a peak in the DLPFC (MNI 44, 28, 32) and DMPFC (MNI 8, 52, −20). In the DLPFC there was reduced activation in the AN group compared with HC in the low-calorie condition. No differences were found in the DMPFC. Thirdly, there was reduced activation to the low-calorie condition in the supramarginal gyrus in the AN group compared to HC. No significant differences were observed in the contrast AN vs AN-R or AN-R vs HC.

### 3.5 Connectivity analyses (PPI)

In the pictures versus baseline contrast across groups, using small volume correction analysis, we identified a significant functional cluster in the anatomical left amygdala, bilateral insula and caudate anatomical masks created from the MNI structural atlas in FSL. No significant activation was seen in the right amygdala. No altered connectivities of the insula were found. There were no significant correlations between relative motion and extracted BOLD signal values in the PPI analysis.

#### 3.5.1 Left amygdala

**Fig. 4:**
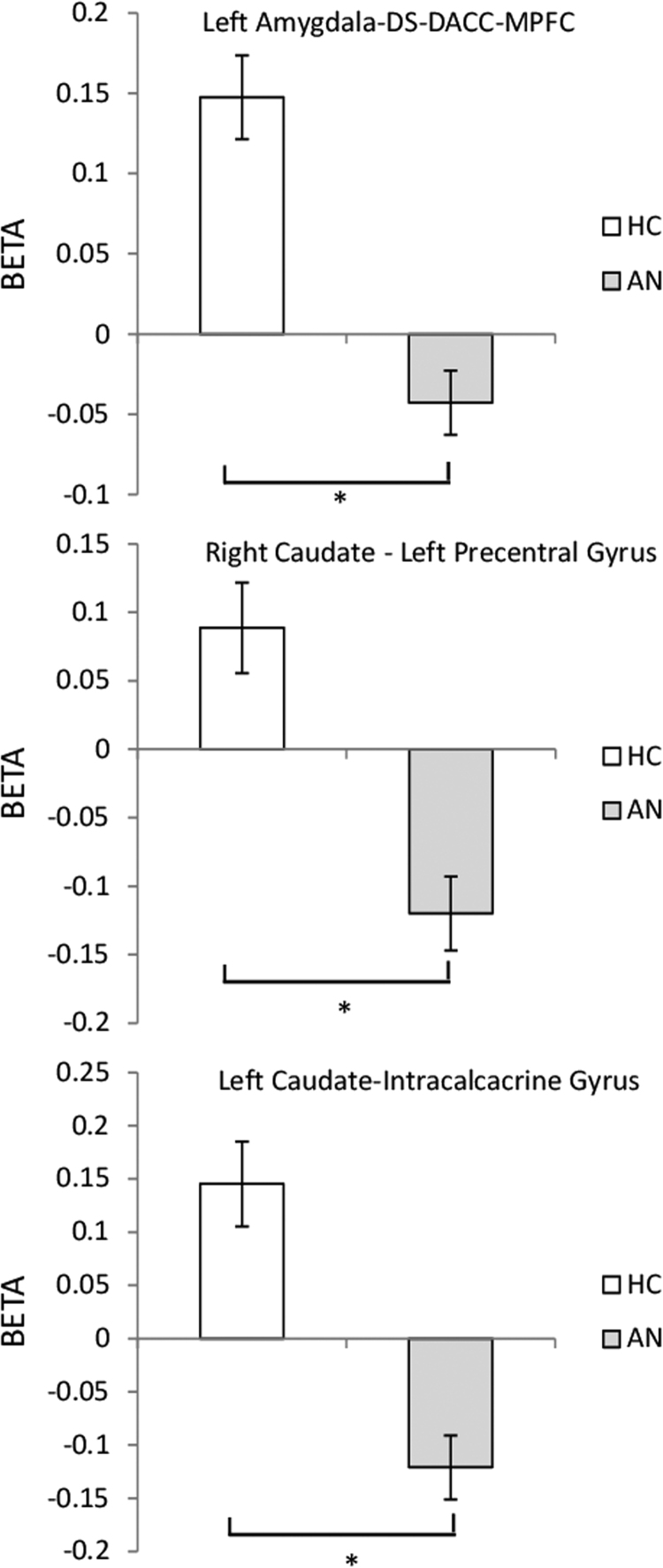
Axial and sagittal images showing: Top: Significantly reduced connectivity between the left amygdala and a cluster extending from the putamen to the anterior cingulate in the AN group compared with HC in the contrast food pictures vs baseline. Middle: Significantly reduced connectivity between the right caudate and the left postcentral gyrus in the AN group compared with HC in the contrast food pictures vs baseline. Bottom: Significantly reduced connectivity between the left caudate and the bilateral intracalcarine - lingual gyri in the AN group compared with HC in the contrast high-calorie food pictures vs baseline.

**Fig. 5:**
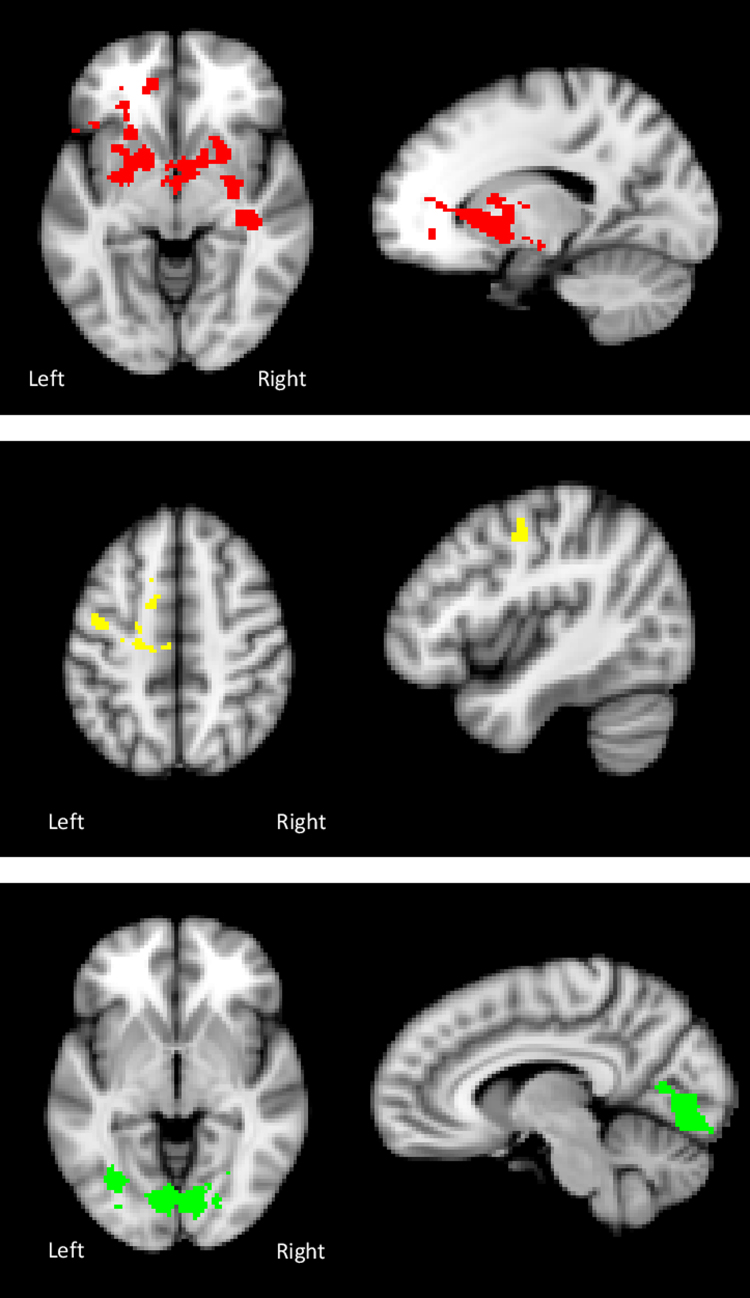
Top: Food pictures vs baseline: Reduced functional connectivity between the left amygdala and the putamen/caudate – dorsal ACC and MPFC in the AN group compared to HC. Middle: Food pictures vs baseline: Reduced functional connectivity between the right caudate and the left postcentral gyrus in the AN group compared to HC. Bottom: High-calorie food pictures vs baseline: Reduced functional connectivity between the left caudate and the bilateral intracalcarine/lingual gyri in the AN group compared to HC.

During food picture blocks (versus baseline), in the AN vs HC contrast, there was a reduced temporal coherence between the left amygdala and a large cluster in the caudate/putamen (dorsal striatum, DS) with subclusters in the dorsal anterior cingulate cortex (DACC) and medial prefrontal cortex (MPFC), see Table 3 and Fig. 4, Fig. 5. In the AN group, there was a significant negative correlation between strength of coupling of the left amygdala and the putamen–anterior cingulate cluster and the YBC-EDs-SRQ past score (r=0.784, *p=*0.003). The greater the severity of the illness in the past (higher YBC-EDs-SRQ past score), the lower the strength of coupling between the two regions. No significant differences in connectivity were observed in the contrast AN vs AN-R or AN-R vs HC.

#### 3.5.2 Right caudate

During food picture blocks (versus baseline), in the AN group compared to HC, the right caudate showed reduced temporal coherence with left postcentral gyrus – juxtapositional lobule cortex see Table 3 and Fig. 4, Fig. 5. No correlation was found between reduced functional connectivity of the right caudate and measures of symptom severity. No significant differences in connectivity were observed in the contrast AN vs AN-R or AN-R vs HC.

#### 3.5.3 Left caudate

A significant calorie × group interaction was found in temporal coherence of the left caudate and the bilateral intracalcarine-lingual gyri. This was driven by decreased connectivity between these regions during the high-calorie condition in the AN group compared to HC see Table 3 and Fig. 4, Fig. 5. No group effect was seen in the low-calorie condition (*p*>0.05). In the AN group, the strength of coupling of the left caudate and the intracalcarine/lingual gyri in the high-calorie condition positively correlated with LOFPQ scores of explicit wanting (r=0.766 *p=*0.006), implicit wanting (r=0.769 *p=*0.006) and explicit liking for high-calorie food (r=0.78 *p=*0.005). No significant differences in connectivity were observed in the contrast AN vs AN-R or AN-R vs HC.

## 4 Discussion

Our findings suggest distinct functional differences in individuals with AN in response to high vs. low-calorie food pictures compared to healthy controls. Consistent with our predictions, and previous research, dysfunction of cortical control and reward regions and those associated with somatosensory processing were observed in AN. Despite widespread GM reduction in the AN group, these results survived correction for GM differences. No differences were observed in the contrasts AN-R vs AN and AN-R vs HC.

Consistent with our first prediction, increased activation was seen in the lateral frontal pole in response to high-calorie pictures in the AN group relative to HCs. There was an opposing decreased response to low-calorie pictures in both this region and the DLPFC, the inverse of what was observed in the HC group. Furthermore, activation of the frontal pole to low-calorie foods negatively correlated with the severity of current ED related obsessions and compulsions (YBC-EDS-SQR past score).

The frontal pole serves as a supervisory attentional control centre (Burgess et al., 2007, Orr et al., 2015) and the lateral division co-activates with regions known to be involved in top-down control, such as the DLPFC, ACC and anterior insula (Gilbert et al., 2010, Orr et al., 2015). This supports our hypothesis that enhanced activation in cognitive control regions is specifically associated with energy-dense foods in AN, which may help explain the underconsumption of high-calorie foods seen in these individuals (Park et al., 2011). Indeed, an inverse pattern of wanting and liking for high-calorie foods was also observed behaviourally, with the AN groups showing decreased scores on these measures compared to controls, corroborating our previous behavioural findings (Cowdrey et al., 2013a).

Whilst this result is in line with the increased cognitive control previously reported in response to food stimuli in AN (Brooks et al., 2011a, Cowdrey et al., 2011), activation in this frontal pole region is a novel finding in AN. The frontopolar cortex (FPC) is central to higher order decision-making and there is evidence that it tracks the relative advantage in favor of switching to a foregone alternative when choices are made voluntarily. Changes in FPC functional connectivity occur when subjects finally decide to switch to an alternative behavior (Rushworth et al., 2012). Increased FPC activity to one choice precedes and predicts choosing the alternative course of action (Boorman et al., 2009). Thus, increased activity in the FPC is thought to reflect the process of accumulating evidence from long term memory in favor of eventually switching to an alternative course of action.

Although our task was not an overtly decision-making one, subjects were asked how much they wanted a particular food, an instruction likely to invoke higher order decision-making processes involving choice, particularly in those with eating disorders. We thus speculate that our finding of differentially increased FPC signal to high-calorie foods in AN may reflect the process of accumulating the evidence in favor of the alternative (not eating or wanting this food). In contrast, paradoxically reduced FPC activity on exposure to low calorie foods (Cowdrey et al., 2011) may reflect the minimal demands on higher order decision-making in those with AN as this is their preferred ‘safe’ food choice. Interestingly this differential effect appears to become more pronounced the more severe the AN pathology is, as it negatively correlates with eating disorder related preoccupations and rituals. Moreover, in line with our behavioural studies (Cowdrey et al., 2013a) the opposite pattern, of increased FPC activity to low as compared to high calorie foods is seen in controls, who generally choose to eat energy dense over low calorie foods. No functional differences were found in the AN-R group, suggesting that whilst a preference for low-calorie food may persist after recovery, alterations in activation of the frontal pole may be specific to the acute stage of AN.

In addition to investigating regional functional differences, we also conducted a PPI analysis to investigate the functional coherence of the insula, amygdala and caudate during the task. No differences were observed in the functional connectivity of the insula, an interesting finding, which is in line with the findings of another group, who also found no group differences in insula connectivity between AN, AN-R and HC groups in a food-cue task (Sanders et al., 2015). The left amygdala showed reduced connectivity with the dorsal striatum (DS), DACC and MPFC in the AN compared to the HC group. This hypoconnectivity was associated with severity of past ED obsessions and compulsions, an intriguing finding, which may represent a scar of the illness or a pre-existing vulnerability marker. Interactions between the amygdala, the striatum, dopaminergic midbrain, and the ACC are thought to modulate satiety, food motivation and reward (Siep et al., 2009; Murdaugh et al., 2012). Reduced connectivity in circuitry responsible for modulating responses to food stimuli in people with AN may allow their inverted food preferences to continue, despite their being in a state of starvation (Cowdrey et al., 2013a).

We also observed differences in the functional coherence of the caudate, which plays a critical role in the habitual and compulsive behavior that characterizes weight-loss behavior in AN (de Wit et al., 2012, Foerde et al., 2015, Sanchez et al., 2015, Taher et al., 2005, Voon et al., 2015). The left caudate showed reduced functional coherence with the postcentral gyrus (the primary somatosensory cortex) in the AN group relative to the HC group. This result, taken together with our finding of reduced activation in somatosensory regions in the AN group when viewing food pictures, furthers our understanding of the neural processes which may underpin the reduced self-reported hunger in the AN group. Finally we saw reduced connectivity between the right caudate and the intracalcarine/lingual gyri, a region responsible for visual memory and the encoding of complex images (Machielsen et al., 2000), in the AN group relative to controls. We speculate that this may, in part, explain the difficulty in integrating visual information seen in AN. Alterations in the connectivity of somatosensory and visual pathways have previously been reported in AN, and linked to the disturbances in body image often reported in AN (Favaro et al., 2012). No differences were observed between AN-R group and the healthy controls, suggesting that alterations in the connectivity of these regions is specific to the acute stage of AN.

Taken together, these findings are consistent with the psychopathology and reported experiences of those with AN. Compulsive and persistent avoidance of high-calorie food in acute AN (Godier and Park, 2014, Godier and Park, 2015b) can continue unchecked by feedback from somatosensory regions regarding body state and hunger, further allowing the relentless pursuit of self-starvation (Park et al., 2012, Park et al., 2014).

As well as some important strengths, this study has notable methodological limitations. The sample size used was small, although heterogeneity was reduced by confining the investigation to purely restrictive AN. Future research may benefit from larger sample sizes, potentially including sufficient sample sizes of both AN and AN-R subtypes, to explore subtype-specific processes. This was a small, exploratory study, and a threshold of Z=2.0 was applied in the analysis. Results do not survive a more conservative threshold of Z=3.0, and this should be borne in mind when interpreting the data.

In this patient population, comorbidities are common and several individuals were on psychotropic medications, which have been shown to alter neural connectivity (Lui et al., 2010, McCabe and Mishor, 2011, van de Ven et al., 2013), so the effects of these, on our results cannot be ruled out. In addition, as no differences were observed between the AN-R and HC groups, it was not possible to determine whether changes in the AN group result from malnutrition or acute illness.

Nevertheless, consistent with our predictions we observed differential activation in the frontal pole in response to high vs. low-calorie food pictures, a novel finding in AN. Decreased activation was also observed in response to both calorie conditions in somatosensory regions, and hypo-connectivity was seen between these areas and reward processing regions. These findings may have translational implications as they suggest possible mechanisms underlying the persistent avoidance of high-calorie food in AN in the face of starvation.

## Financial disclosures/conflicts of interest

CJH has received consultancy fees from Lundbeck, P1vital, Astra Zeneca and Servier. She is a company director of Oxford Psychologists Ltd and holds shares in the same company. CJH has received grant funding from UCB, J&J, Astra Zeneca, Lundbeck and Sunovion.

JCS, LRG, AR and RJP report no biomedical financial interests or potential conflicts of interest.

## Figures and Tables

**Table 1:** Demographics, mood and anxiety questionnaire, and eating disorder scores in the three groups (*M*±*SD*, one-way *ANOVA P*-scores). Post-hoc differences between AN and HC indicated by *, between both AN and HC and AN-R and HC indicated by **. Where two groups were compared, P-Score is from independent samples T tests.

	***Healthy controls***	***Recovered***	***Patients***	***P-score***
**Sociodemographic data**	(*N=*16)	(*N=*14)	*N*=(12)	
**Age**	24.3±5.7	27±6.5	29.4±6.0	0.092
**BMI**	21.2±2.0	20.9±1.6	15.4±1.9	<0.001^*^
**NART**	109.6± 6.8	114.6±6.8	114.7±8.3	0.159
**Age of onset of AN (years)**	N/A	16.5±2.1	20.1±5.9	0.04
**Duration of AN (years)**	N/A	5.8±4.2	10.3±5.2	0.02
**EDE**	0.2±0.2	0.7±0.6	2.8±1.6	<0.001^*^
**VAS ‘thinness’**	47.5±15.3	54.8±11.2	52.0±27.9	0.6
**VAS ‘fear of weight gain’**	31.9±19.9	54.9±24.1	79.4±19.9	<0.001^**^
**VAS ‘hunger’**	71.5±20.0	54.8±11.2	52.0±27.9	0.016^*^
**STAI – State**	26.9±6.532.9±9.7	34.4±5.445.2±10.6	51.2 ± 15.561.7±14.0	<0.001^*^
**STAI - Trait**	32.9±9.7	45.2±10.6	61.7±14.0	<0.001**
**BDI**	3.3±4.5	6.4±6.4	30.3±18.7	<0.001*
***YBC-EDS- SRQ Current***	N/A	3.8±3.9	15.2±7.7	<0.001
***YBC-EDS-SRQ Past***	N/A	23.8±5.3	23.3±5.6	0.82

Note: BMI=Body Mass Index; NART=National Adult Reading Test; STAI=State Trait Anxiety Inventory; BDI=Beck Depression Inventory; EDE=Eating Disorder Examination; VAS=Visual Analogue Scales; YBC-EDS-SRQ=Yale-Brown-Cornell Eating-Disorder-Scale Self-Report Questionnaire.

**Table 2:** (A) Areas of significant difference in % signal change viewing of food pictures vs baseline across groups. (B) Areas of significant difference in % signal change to high vs low calorie pictures. Peak clusters are highlighted in bold, followed by subclusters. * DLPFC 10 mm sphere.

(A) Group differences: pictures vs baseline									
	**Side**		**Cluster size (voxels)**	**MNI (x, y, z)**	**Z-score**		**Group difference**	***t*****value**	***p*****value**
**Postcentral Gyrus**	R		4214	40 −30 58	4.47		AN<HC	3.9	0.001
Precuneus				2 −66 26	4.4				
**Superior parietal lobule**	L		1516	−40 −44 56	3.94		AN<HC	2.9	0.007
Postcentral gyrus				48 −24 58	3.85				
(B) Group differences, high calorie (HCal) vs low calorie (LCal) pictures									
		**Side**	**Cluster size (voxels)**	**MNI (x, y, z)**		**Z-score**	**Group difference**	***t*****value**	***p*****value**
**Frontal pole**		R	798	28 64 0		4.51	AN>HC (HCal)	−3.1	0.004
							AN<HC (LCal)	3.0	0.006
VMPFC (small cluster)				8 52 −20		3.85			
**DLPFC**		R	3735	44 28 32		4.26	AN<HC (LCal)	2.5*	0.018
DMPFC				8 52 44		3.95			
**Supramarginal/angular gyrus**		R	3622	62 −48 16		4.38	AN<HC (LCal)	4.1	<0.001
Precuneus/				2 −66 32		4.02			
Lateral Occipital Cortex				56 −68 16		3.8			

*Note:* P>B=pictures vs baseline; H>L=High vs low calorie; DLPFC=dorsolateral prefrontal cortex; DMPFC=dorsomedial prefrontal cortex, VMPFC=ventromedial prefrontal cortex L=left; R=right; MNI=Montreal Neurological Institute.

**Table 3:** Psychophysiological interactions. (A) Areas of significant difference in BOLD response viewing of food pictures compared with baseline across groups. (B) Areas of significant difference in BOLD response High vs Low calorie blocks. MNI coordinates refer to the peak activation voxel of the cluster and main sub-regions within the same cluster (significant group differences bold-printed).

(A) Pictures vs baseline/group effects							
Decreased coherence with the **left amygdala** in AN group when viewing pictures (P>B)							
	**Side**	**Cluster size (voxels)**	**MNI (x, y, z)**	**Z-score**	**Group difference**	***t*****value**	***p*****value**
**Putamen/Caudate-DACC, MPFC**	L	3728	−16 6 −6	3.85	AN<HC	4.6	<0.001
Decreased coherence with the **right caudate** in AN group when viewing pictures (P>B)							
	**Side**	**Cluster size (voxels)**	**MNI (x, y, z)**	**Z-score**	**Group difference**	***t*****value**	***p*****value**
**Precentral- middle frontal gyrus**	L	627	−42 −8 48	3.99	AN<HC	3.8	0.001
Juxtapositional lobule cortex	L		−12 4 54	2.91			
(B) High vs low calorie (H>L), AN>HC							
Decreased coherence with the **left caudate** in AN group when High calorie food pictures (H>L)							
	**Side**	**Cluster size (voxels)**	**MNI (x, y, z)**	**Z-score**	**Group difference**	***t*****value**	***p*****value**
**Intracalcacrine -lingual gyrus**	R	3311	8 −80 −4	3.96	AN<HC	4.1	<0.001
**Lingual – occipital fusiform gyrus**	L		−16 −76 −14	3.88			

*Note:* DACC=dorsal anterior cingulate cortex, MPFC=medial prefrontal cortex, VMPFC=ventromedial prefrontal cortex L=left; R= right; MNI=Montreal Neurological Institute.

## Footnotes

Appendix A Supplementary data associated with this article can be found in the online version at doi:10.1016/j.pscychresns.2016.10.004.
