# Supplementary material for: Differential activation of the frontal pole to high vs low calorie foods: The neural basis of food preference in Anorexia Nervosa?
Source: Psychiatry Res. 2016 Dec 30;258:44–53. doi: 10.1016/j.pscychresns.2016.10.004 (PMC5146322; doi:10.1016/j.pscychresns.2016.10.004)
Supplement: Supplementary file 1 — Supplementary material [file mmc1.docx]

**Figure 6:**

**Top:** Implicit wanting: Reduction in implicit wanting of high calorie foods in both the AN-R group and AN group in the high calorie condition compared to HC.

**Middle:** Explicit wanting: Reduction in explicit wanting of high calorie foods in both the AN-R group and AN group in the high calorie condition compared to HC.

**Bottom:** Explicit Liking: Reduction in liking of high calorie foods in both the AN-R group and AN group in the high calorie condition compared to HC.

Increased liking of low calorie foods in both the AN-R group and AN group in the high calorie condition compared to HC.

**Figure 7:** GM reduction in the AN group compared to HC in a) the frontal pole, b) putamen and thalamus, c) left supramarginal gyrus/superior parietal lobule

**Figure** Reduction in GM in.


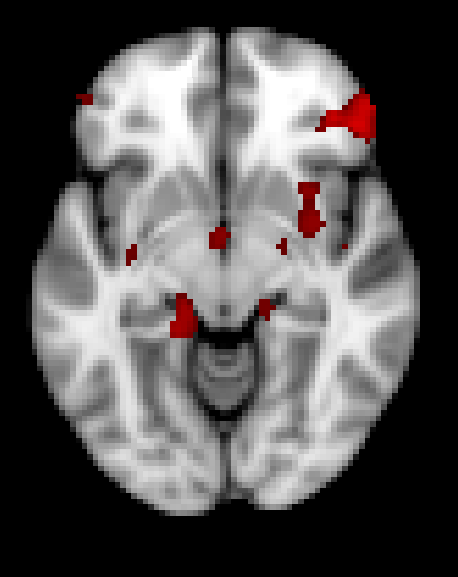

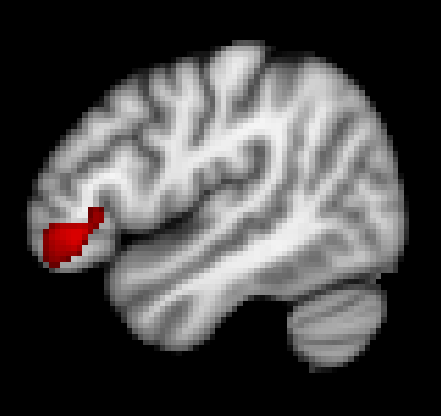

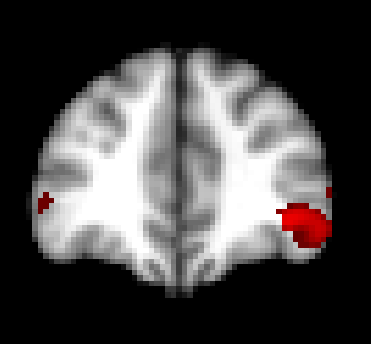


a

Left

Right


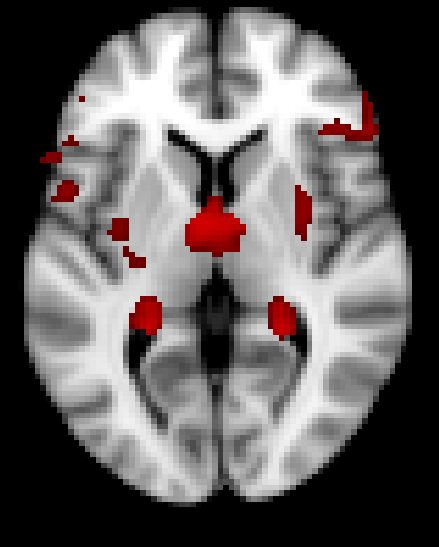

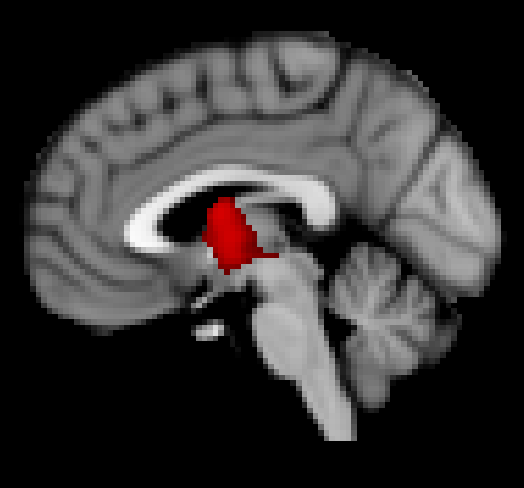

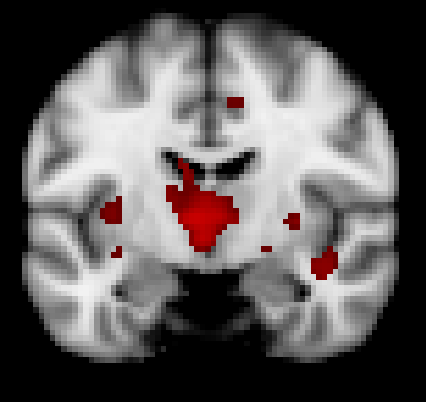


b


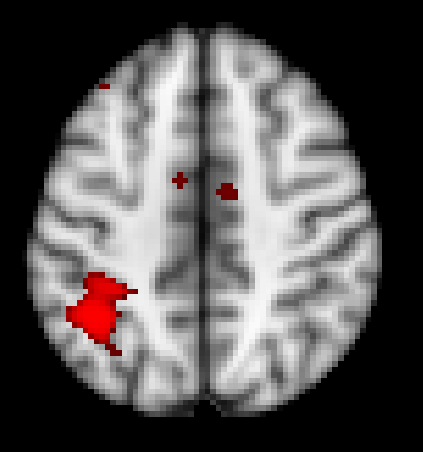

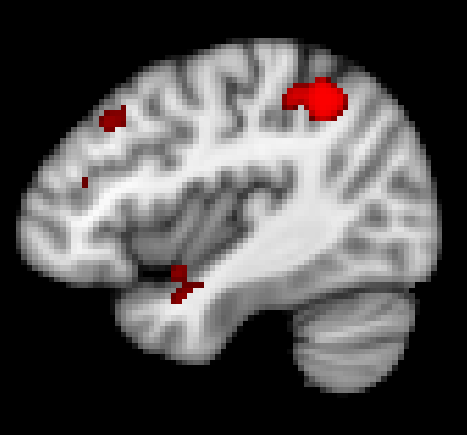

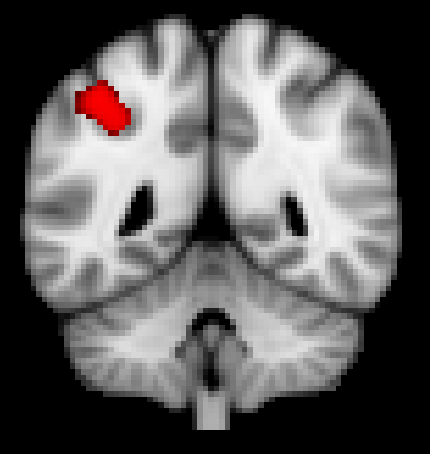


c

**Figure 7 d:** GM reduction in the left supramarginal gyrus/superior parietal lobule in the AN-R group vs HC (yellow) overlaid GM reduction in AN group (red)

c

d


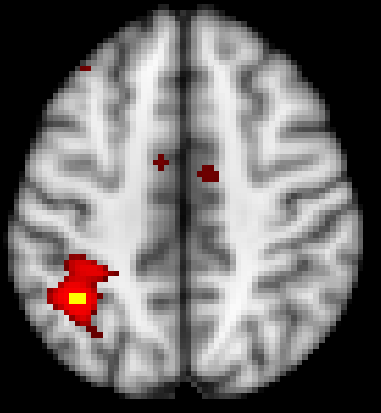

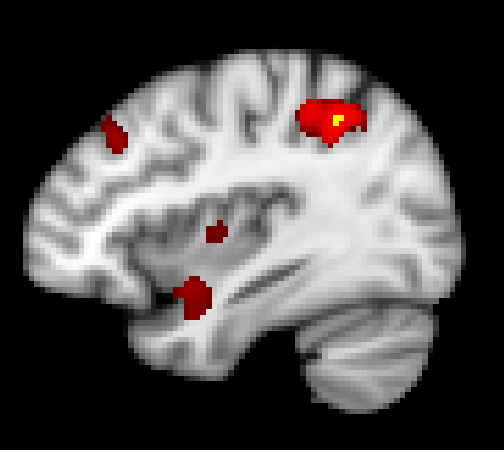

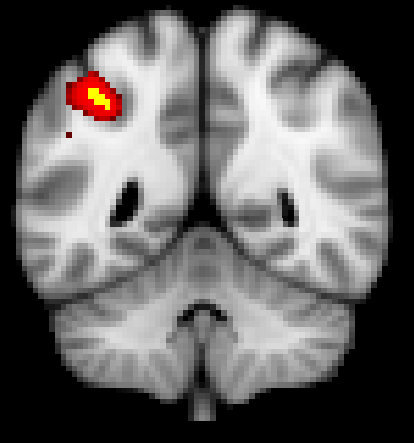


d

Table 5. Explicit wanting and liking and implicit wanting across the groups

|  | Implicit wanting | | | | Explicit wanting | | | | Explicit liking | | | |
| --- | --- | --- | --- | --- | --- | --- | --- | --- | --- | --- | --- | --- |
|  | High calorie | | Low calorie | | High calorie | | Low calorie | | High calorie | | Low calorie | |
|  | *M* | *SD* | *M* | *SD* | *M* | *SD* | *M* | *SD* | *M* | *SD* | *M* | *SD* |
| HC | 69.6 | 21.8 | 54.0 | 18.5 | 71.7 | 21.1 | 51.2 | 17.1 | 14.7 | 20.4 | -4.7 | 20.4 |
| AN-R | 37.2 | 20.2 | 40.6 | 16.8 | 40.3 | 19.9 | 51.3 | 21.1 | -8.7 | 20.5 | 8.7 | 20.5 |
| AN | 26.4 | 25.4 | 37.9 | 22.1 | 34.3 | 26.8 | 53.9 | 25.5 | -20.6 | 25.1 | 20.6 | 25.1 |

*Note*. HC = Healthy control; AN-R = Recovered anorexia nervosa; AN = Current anorexia nervosa.

Table 6: Global GM, WM and CSF volumes expressed as a percentage of total brain volume in the three groups (*M* ± *SD*, one-way *ANOVA*  *P*-scores).

|  |  | *Healthy Controls*  *(N=16)* | *Recovered*  *(N=14)* | *Patients*  *(N=12)* | *P-score* |
| --- | --- | --- | --- | --- | --- |
|  |  |  |  |  |  |
|  | Percentage WM | 37.5 ± 1.4 | 37.8 ± 1.1 | 37.1 ± 1.3 | 0.45 |
|  | Percentage GM | 43.0 ± 1.1 | 42.2 ± 1.6 | 40.0 ± 4.5 | 0.013 |
|  | Percentage CSF | 19.5 ± 1.6 | 20.0 ± 2.2 | 22.2 ± 2.3 | 0.004 |

**Food Pictures Task**

Stimuli were 40 high resolution (1034x768), standardised digital colour photographs of foods divided equally into high-calorie and low-calorie categories. These images were provided by L. Charbonnier of the Image Sciences Institute, UMC Utrecht, and created as part of the Full4Health project (www.full4health.eu), funded by the European Union Seventh Framework Program (FP7/2007‐2013) under grant agreement nr. 266408.

**Analysis of Structural MRI**

Whole brain analysis was carried out using a voxel-based morphometry-style analysis (FSL-VBM) ([Douaud et al., 2007](#_ENREF_20)) using default settings as described at [www.fmrib.ox.ac.uk/fsl/fslvbm/](http://www.fmrib.ox.ac.uk/fsl/fslvbm/). Brain extraction and tissue-type segmentation were performed and resulting GM partial volume images were aligned to standard space using non-linear (FNIRT) registration tools. The resulting images were averaged, modulated and smoothed with an isotropic Gaussian kernel of 5 mm FWHM to create a study-specific template, and the GM images were re-registered to this, including modulation by the warp field Jacobian. Finally, voxel-wise GLM was applied using permutation-based non-parametric testing (5000 permutations), clusters were determined by Z > 2.3 and then a family-wise-error (FWE) corrected cluster significance threshold of p < 0.05 was applied to the suprathreshold clusters. Structural MRI was acquired at the end of the two functional MRI sequences.

Furthermore, for each subject, GM, white matter and cerebrospinal fluid volumes were derived using a brain tissue segmentation tool (FAST) and computed as a percentage of total brain volume. This was compared between groups and GM volume in the AN group was correlated with BMI, age, lowest BMI, duration of disease and YBCEDs scores. Statistical analyses of non-imaging variables were carried out using SPSS software (SPSS, Inc., Chicago IL) version 22.0. Threshold for statistical significance was set to p<0.05.

**Figure 8. Results from the main task analysis, with values from the AN-R group added for illustrative purposes.**

In the food pictures vs baseline condition, reduced activation was observed in a) the right superior parietal lobule-precentral gyrus and b) the left precentral gyrus-precuneus in the AN group compared to HC.

High vs Low-calorie food pictures.

c) Frontal pole: In the high-calorie condition there was an increased response in the AN group compared to HC. In the low-calorie condition there was a decreased response in the AN group compared to HC.

d) In the low-calorie condition there is a decreased response in the DLPFC 10mm sphere ROI in the AN group compared to HC.

e) In the low-calorie condition there is decreased response in the supramarginal/lingual gyri in the AN group compared to HC.

Frontal Pole

**Figure 9. Results from the PPI analysis, with values from the AN-R group added for illustrative purposes.**

**Top** Food pictures vs baseline: Reduced functional connectivity between the left amygdala and the putamen/caudate – dorsal ACC and MPFC in the AN group compared to HC.

**Middle**  Food pictures vs baseline: Reduced functional connectivity between the right caudate and the left postcentral gyrus in the AN group compared to HC.

**Bottom** High-calorie food pictures vs baseline: Reduced functional connectivity between the left caudate and the bilateral intra-calcarine/lingual gyri in the AN group compared to HC.

*[Type a quote from the document or the summary of an interesting point. You can position the text box anywhere in the document. Use the Drawing Tools tab to change the formatting of the pull quote text box.]
